# The Law as to Death Certificates

**Published:** 1898-04-30

**Authors:** 


### THE LAW AS TO DEATH CERTIFICATES.

Commenting on a recent case in which a medical practitioner thought himself entitled to withhold a certificate of the cause of death, a correspondent writes as follows in regard to the law on the subject:—It seems almost inconceivable that any medical man could be so ignorant of the duties imposed upon him by Act of Parliament as to imagine that he could withhold a certificate of the cause of death until a payment was made. The wording of the Act, so far as it relates to the subject, is printed on the back of every certificate form, and is absolutely positive. The practitioner "shall sign and give . . . a certificate." No exemption is made so long as the doctor has such "knowledge and belief" as to the cause of the death as to enable him to certify it. A good deal of misconception seems to exist in the mind of the public, and even among medical men and coroners, as to the power of a doctor to refuse a certificate. There can be no doubt that, according to the strict wording of the Act, a medical man has no right to refuse a certificate in any case, even in a case of accident or murder, so long as he knows the cause of death and has attended the patient during his last illness. All he has to make sure of is that the certificate is true and bears upon the face of it the fact that injury, or poison, or what not, was the cause of death, however remotely. The duty of refusing the burial certificate is thrown on the registrar, not on the doctor. The general practice, however, is no doubt that which is recommended in Mr. Braxton Hicks' little book on the subject—to decline to give a certificate to the friends and refer them at once to the coroner's officer, but this is not in the Act.

